# Supplementary material for: Enhancing sub-bandgap external quantum efficiency by photomultiplication for narrowband organic near-infrared photodetectors
Source: Nat Commun. 2021 Jul 15;12:4259. doi: 10.1038/s41467-021-24500-2 (PMC8282846; doi:10.1038/s41467-021-24500-2)
Supplement: Supplementary file 1 — Supplementary Information [file 41467_2021_24500_MOESM1_ESM.pdf]

Supplementary Information for:

Enhancing Sub-Bandgap External Quantum Efficiency by Photomultiplication for Narrowband Organic Near-Infrared Photodetectors

Jonas Kublitski[1*], Axel Fischer[1], Shen Xing[1], Lukasz Baisinger[1], Eva Bittrich[2], Donato Spoltore[1], Johannes Benduhn[1], Koen Vandewal[3], Karl Leo[1,4*]

[1]Dresden Integrated Center for Applied Physics and Photonic Materials (IAPP) and Institute for Applied Physics, Technische Universität Dresden, Nöthnitzer Str. 61, 01187 Dresden, Germany

[2]Leibniz-Institut für Polymerforschung Dresden e.V., Hohe Str. 6, 01069 Dresden, Germany

[3]Instituut voor Materiaalonderzoek (IMO), Hasselt University, Wetenschapspark 1, BE-3590, Diepenbeek, Belgium

[4]Center for Advancing Electronics Dresden (cfaed), Technische Universität Dresden, 01062 Dresden, Germany

*Corresponding authors: jonas.kublitski@tu-dresden.de, karl.leo@tu-dresden.de

Contents

1 Supplementary Figures 3
2 Supplementary Tables 6

Supplementary References 6

1 Supplementary Figures

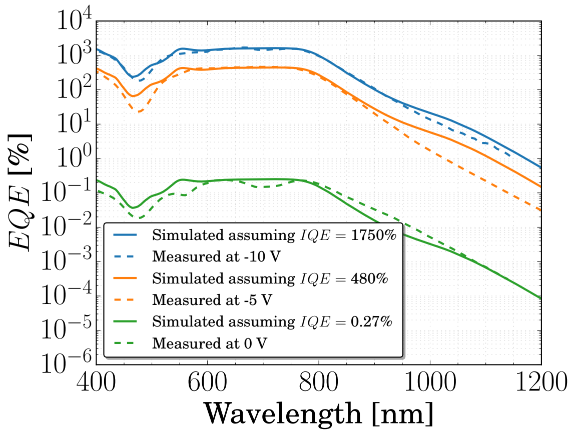

Supplementary Figure 1: Internal quantum efficiency (IQE) calculation. External quantum efficiency (EQE) measured at different biases (solid lines) and simulated EQE using transfer matrix method (TMM) (dashed lines). The absorption spectra is simulated by TMM and the simulated EQE spectra are matched to the experimental ones by assuming IQE values as indicated in the legend for different biases. Even though EQE is lower than 100% in the charge-transfer state absorption range, photomultiplication happens nearly with the same efficiency as in the main absorption region. At -10 V the device reaches an IQE of 1750%.

(a) **(b)**

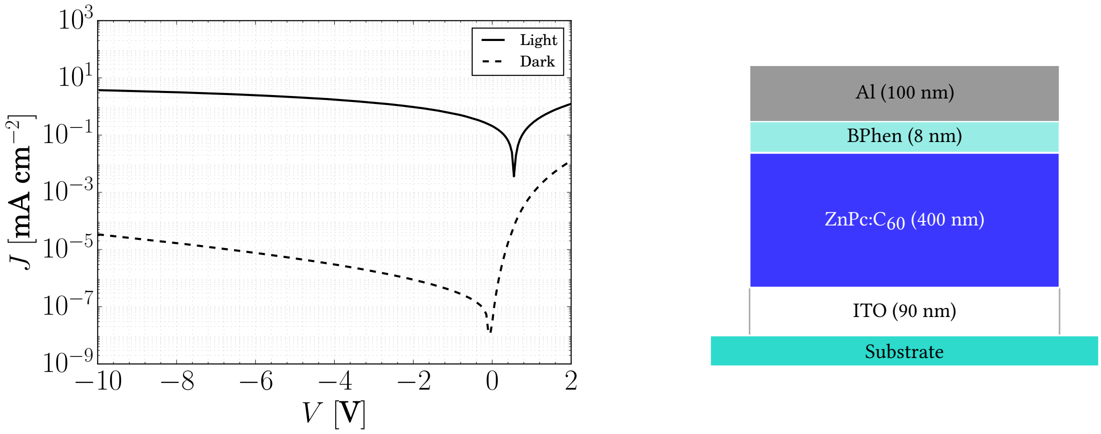

Supplementary Figure 2: pin-diode. (a) JV curves under dark and under $100\,\mathrm{mW\,cm^{-2}}$ illumination. **(b)** structure of the pin-diode.

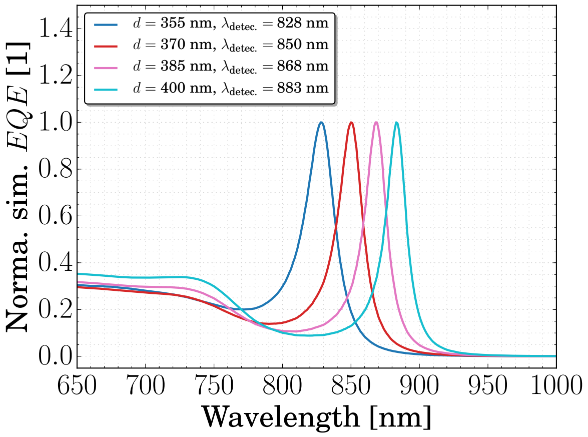

Supplementary Figure 3: TMM simulation of narrowband devices. Normalized simulated EQE for four different thicknesses (d) of the optical microcavity, leading to four detection wavelengths ($\lambda_{\text{detec.}}$), as presented in the main text.

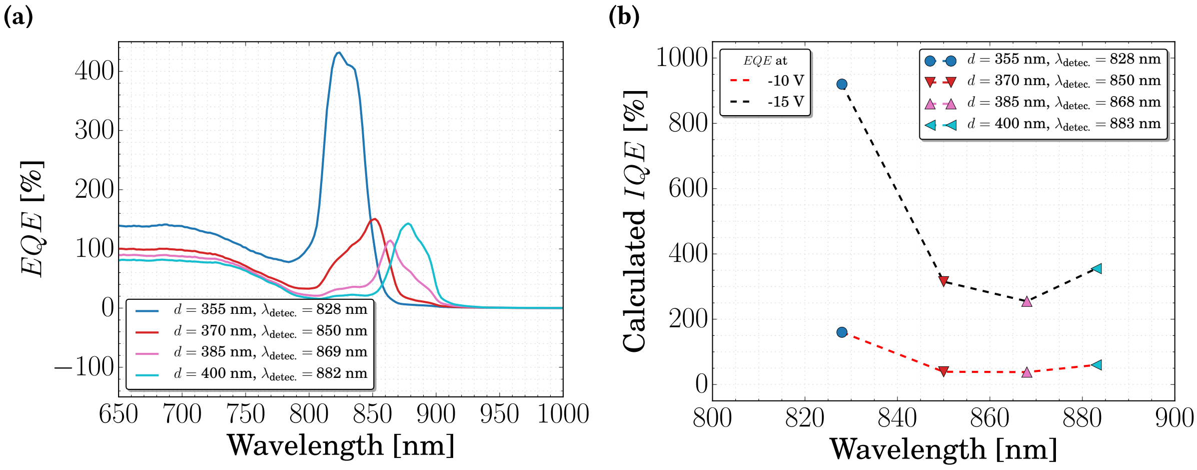

Supplementary Figure 4: EQE and IQE of narrowband devices. **(a)** EQE of narrowband devices measured at -15 V. **(b)** Estimated IQE at -15 V (dashed black line) and -10 V (red dashed line). At -15 V both **(a)** EQE and **(b)** IQE of all narrowband devices are higher than 100%, demonstrating that photomultiplication is achieved for these devices. In **(b)**, dashed lines are guide to the eye.

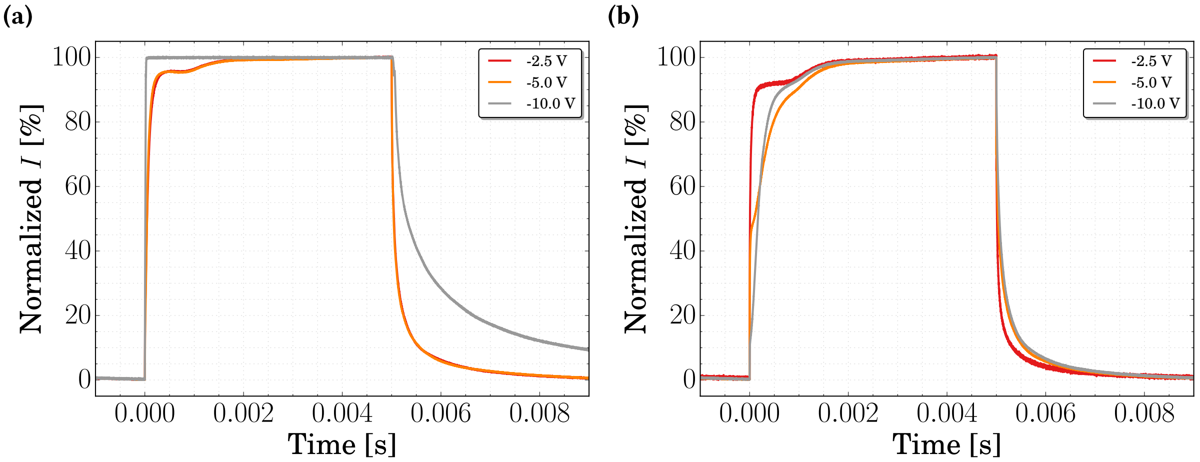

Supplementary Figure 5: Transient photocurrent of (a) broad- and (b) narrowband PM-OPDs. For all measurements, 100 Hz pulse signal was used to probe the white LED, except for the broadband device at -10 V, where 50 Hz was used due to the long decay time. Switching-on and -off time constants determined as the time the device response takes to rise from 10% to 90% (on) and to fall from 90% to 10% (off) of its maximum value. The time constants are summarized in Supplementary Table 1.

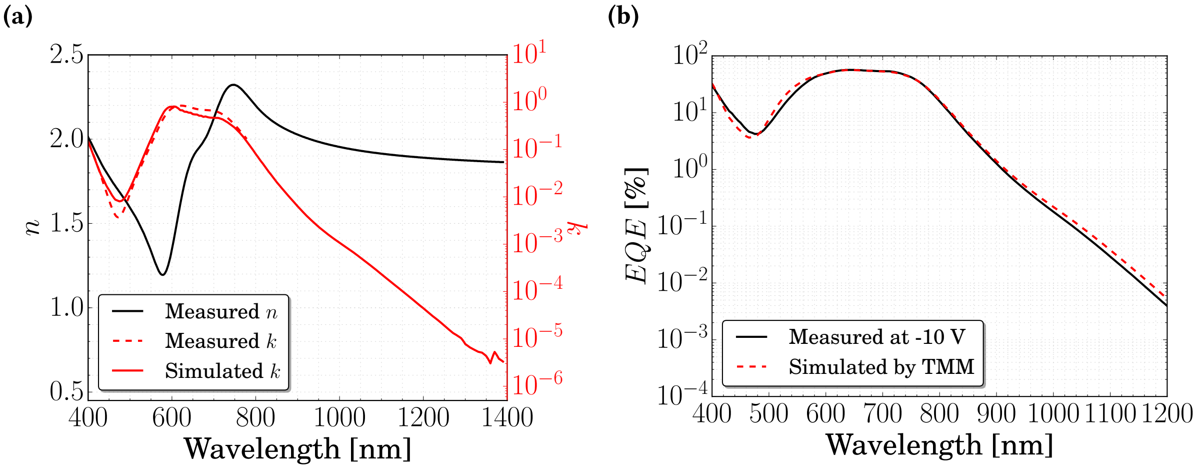

Supplementary Figure 6: n, k-values of ZnPc:C_{60} (3 wt%). (a) In-plane n-values are obtained from variable-angle spectroscopic ellipsometry as described in the main text. The absorption in the CT state absorption region is very weak, hindering the modeling of k-values from spectroscopic ellipsometry. As we are mainly interested in this region, we derived k-values as described by Kaiser *et al.*[1]. The method requires device parameters such as EQE spectrum, which is obtained from a pin-diode with the same structure shown in Supplementary Figure 2b, but with active layer thickness of 100 nm. Since the architecture of the pin-diode is not optimized, the EQE is measured at -10 V to ensure that all photogenerated charges are extracted. Additionally, n, k-values of all other layers in the device must be provided, which were obtained by spectroscopic ellipsometry. In **(a)** the simulated k-values (red solid line) are compared to the measured k-values (dashed line) in the visible range. A good agreement is achieved, indicating that the simulated k-values of ZnPc:C_{60} (3 wt%) according to Kaiser *et al.*[1] are properly derived. **(b)** Measured EQE at -10 V of the pin-diode compared to the TMM simulated spectrum of the same device using the n, k-values from **(a)**.

2 Supplementary Tables

Supplementary Table 1: Speed of broad- and narrowband PM-OPDs. The transient photocurrent measurements are shown in Supplementary Figures 5a and 5b, respectively. The switching-on and -off time constants of PM-OPDs are determined as the time the device takes for the response signal to rise from 10% to 90% and to fall from 90% to 10% of its maximum value, respectively.For all measurements, 100 Hz pulse signal was used to probe the white LED, except for the broadband device at -10 V, where 50 Hz was used due to the long decay time. The duration of the light pulse was long enough to reach the steady-state (5 ms), the time at which the curves are normalized. The known relation $f_{-3\,\mathrm{dB}} \simeq 0.35t_{\mathrm{on}}^{-1}$ is used to calculate the cut-off frequencies[2].

	Broadband			Narrowband (843 nm)		
Applied bias [V]	t_{on} [µs]	$f_{-3\,\mathrm{dB}} \simeq 0.35t_{\mathrm{on}}^{-1}$ [kHz]	t_{off} [µs]	t_{on} [µs]	$f_{-3\,\mathrm{dB}} \simeq 0.35t_{\mathrm{on}}^{-1}$ [kHz]	t_{off} [µs]
-2.5	63	5.55	280	135	2.59	326
-5.0	541	0.65	539	941	0.37	613
-10.0	556	0.63	597	18	19.44	667

Supplementary Table 2: Process parameters for the vacuum deposition of PM-OPDs.

Materials	Broadband				Narrowband			
	Concentration [wt%]	Thickness [nm]	Rate [$Å\,s^{-1}$]	Vacuum chamber pressure [mbar]	Concentration [wt%]	Thickness [nm]	Rate [$Å\,s^{-1}$]	Vacuum chamber pressure [mbar]
MoO_3	–	–	–	–	100	3	0.2	1.5×10^{-7}
Ag	–	–	–	–	100	25	0.6	2.1×10^{-6}
MeO-TPD	–	–	–	–	100	10	0.5	4.0×10^{-7}
ZnPc	97	388	0.5	2.4×10^{-7}	97	variable	0.5	$\approx 10^{-7}$
C_{60}	3	12	0.015	2.4×10^{-7}	3	variable	0.015	$\approx 10^{-7}$
HATNA-Cl_6	100	10	0.5	2.7×10^{-7}	100	10	0.4	3.2×10^{-7}
Al	100	100	0.5	2.2×10^{-6}	–	–	–	–
Ag	–	–	–	–	100	100	1.0	1.8×10^{-6}

Supplementary References

1. Kaiser, C., Zeiske, S., Meredith, P. & Armin, A. Determining Ultralow Absorption Coefficients of Organic Semiconductors from the Sub-Bandgap Photovoltaic External Quantum Efficiency. *Advanced Optical Materials* **8**, 1901542 (2020). DOI: 10.1002/adom.201901542.

2. Klompenhouwer, M. A. 51.1: Temporal impulse response and bandwidth of displays in relation to motion blur. *SID Symposium Digest of Technical Papers* **36**, 1578–1581 (2005). DOI: 10.1889/1.2036313.
